# A Multi-Omics Perspective on *Tritrichomonas foetus*: From Genomics to Future Directions

**DOI:** 10.3390/ijms26178343

**Published:** 2025-08-28

**Authors:** Joanna Dąbrowska, Jacek Sroka

**Affiliations:** 1 Department of Virology and Viral Diseases of Animals, National Veterinary Research Institute, 24-100 Puławy, Poland; 2 Department of Parasitology and Invasive Diseases Bee Diseases and Aquatic Animal Diseases, National Veterinary Research Institute, 24-100 Puławy, Poland; jacek.sroka@piwet.pulawy.pl

**Keywords:** *Tritrichomonas foetus*, genomics, transcriptomics, proteomics, future perspectives

## Abstract

*Tritrichomonas foetus* is an anaerobic flagellated protozoan that infects multiple animal hosts, primarily cattle and cats, with occasional isolation from pigs. It causes bovine trichomonosis, a venereal disease associated with infertility, abortion, and economic losses in cattle herd. In cats, *T. foetus* infects the gastrointestinal tract, causing chronic diarrhea which can be difficult to treat. Despite its broad impact, the pathogen is difficult to control because it evades immune responses and persists in host tissues. Recent advances in omics technologies, including genomics, transcriptomics, and proteomics, have contributed to a better understanding of the parasite’s genetic structure, virulence, drug resistance mechanisms, and metabolic pathways. These findings have identified potential drug targets and paved the way for targeted therapies. However, the biology, pathogenicity, and host interactions with *T. foetus* are still not fully understood, and many aspects of its life cycle and molecular mechanisms remain to be elucidated. This review summarizes the latest omics research on *T. foetus*, highlighting its genetic diversity and host-specific adaptations, and outlines the gaps in our understanding.

## 1. Introduction

*Tritrichomonas foetus*, a single-celled, anaerobic flagellate parasite belonging to the Parabasalia phylum, represents a pathogen of considerable veterinary and economic significance. This protozoan parasite colonizes various species of animal hosts, leading to markedly different clinical signs [1]. In cattle, *T. foetus* causes bovine trichomonosis, leading to reproductive losses due to infertility, embryonic loss, and abortion. Infected bulls are often asymptomatic carriers and may require removal from breeding herds. In cats, the parasite infects the gastrointestinal tract, causing chronic, persistent diarrhea that is often resistant to treatment and prone to relapse. However, in swine, from which it is also occasionally isolated, *T. foetus* is generally regarded as a commensal organism, typically residing in the nasal cavity, stomach, and intestines without inducing noticeable clinical symptoms [2]. Despite its profound and varied impact on animal health and the agricultural economy, *T. foetus* poses persistent challenges to effective control. Its ability to establish persistent infections by modulating or evading host immune responses, coupled with the documented emergence of resistance to standard antiprotozoal therapies, complicates disease management and indicates the need for new intervention strategies [3,4]. The distinct modes of infection exhibited by the parasite in different hosts, ranging from reproductive tract colonization to gastrointestinal habitation, highlight the need to understand the molecular basis of its host-specific adaptations. A major unresolved aspect of *T. foetus* biology is its remarkable ability to inhabit three distinct animal species—cats, cattle, and pigs—colonizing different anatomical sites within each host. Elucidating the molecular mechanisms that govern this host specificity and pathogenic versatility remains an important research question in modern parasitology.

In recent years, the “omics” revolution has provided unprecedented, new tools to address these complex biological questions. This suite of high-throughput technologies—encompassing genomics, transcriptomics, and proteomics—facilitates a holistic, systems-level investigation of organisms that was previously impossible. By interrogating the parasite’s genetic blueprint, genomics reveals not only its evolutionary history but also the full repertoire of genes potentially associated with virulence, drug resistance, and metabolic adaptation, including large gene family expansions [5]. Capturing the dynamism of the parasite’s response to its environment, transcriptomics moves beyond the static genome to quantify gene expression, clarifying how *T. foetus* remodels its physiology and engages in a molecular conversation with its host. Finally, proteomics provides a direct window into its functions by analyzing the protein effectors that mediate host-parasite interactions and execute cellular processes, providing insight into this parasite’s functions, including post-translational modifications invisible to other omics approaches. Recent use of these technologies in *T. foetus* research has generated data on the molecular mechanisms underlying immune evasion, drug resistance, and host adaptation [6].

This review aims to synthesize the latest advancements in omics research on *T. foetus*, highlighting its genetic diversity, host-specific adaptations, and the critical gaps in our current knowledge. By integrating multiple omics datasets, we seek to gain a holistic perspective on the parasite’s biology and its intricate interactions with its hosts. In doing so, we will chart the journey from early genomic drafts to chromosome-level assemblies, explore the transcriptomic signatures that define host adaptation, and dissect the proteomic data that reveals the parasite’s functional arsenal, providing new avenues for developing the next generation of effective diagnostics, targeted therapies, and preventive measures in veterinary medicine. In addition to advancing basic knowledge, multi-omics insights offer tangible opportunities for the development of new diagnostics, targeted therapies, and potential vaccine candidates, bridging the gap between molecular understanding and clinical practice.

## 2. From Drafts to Chromosomes: The Evolving Landscape of *Tritrichomonas foetus* Genomics

The genomic exploration of *T. foetus* has been shaped by technical and biological obstacles. Charting the genetic blueprint of this parasite has historically been hindered by its inherent structural complexity, most notably a vast amount of repetitive DNA—primarily transposable elements (such as DNA transposons and retrotransposons) and satellite repeats—that renders the de novo genome assembly exceptionally challenging [7]. Limited research focus and biological complexity have contributed to a relatively sparse genomic dataset for this pathogen. Several studies, ranging from the initial draft genome of the K strain to the recent KV-1 assembly using Hi-C technology, have progressively clarified the genetic architecture of this protozoan (Table 1) [7,8,9].

### 2.1. Genome Assembly and Structure: Navigating a Labyrinth of Repeats

Analyses of the *T. foetus* genome have been hindered by its highly repetitive sequence content, similar to that observed in *Trichomonas vaginalis*. The genome, estimated at approximately 161 Mb, contains a high repeat content of ~62%, which has posed a challenge for available sequencing technologies. This resulted in a highly fragmented draft assembly comprising 3730 contigs, with the resulting scaffolds covering less than half of the predicted genome size, resulting in the structural organization being largely unresolved. This fragmentation impaired gene prediction, leaving nearly half (47%) of predicted proteins uncharacterized as “hypothetical” [9].

These challenges were not unexpected, as the *T. vaginalis* genome is notorious for its massive expansion driven by transposon-related elements, its paucity of introns, and its atypical transcriptional regulation centered on the initiator (Inr) element rather than a canonical TATA box [8].

The application of annotation pipelines not optimized for such eukaryotic idiosyncrasies, such as the System for Automated Bacterial Integrated Annotation (SABIA), likely introduced significant artifacts into the early *T. foetus* drafts, with a substantial risk of misidentifying genes and splice sites [9]. SABIA, designed for prokaryotic genomes lacking introns and organized in operons, does not support essential eukaryotic features such as exon–intron boundaries, alternative splicing, and untranslated regions (UTRs), making it unsuitable for the accurate annotation of eukaryotic genomes [10,11].

A major improvement occurred with the chromosome-level assembly of the *T. foetus* KV-1 strain. By integrating short-read, long-read, and Hi-C scaffolding data, this multi-platform strategy successfully navigated the complex repeat landscape. This effort culminated in a high-fidelity 148 Mb reference genome where ~78% of the assembly was anchored into five chromosome-scale super-scaffolds. The contiguity improved, with scaffold N50 increasing to approximately 22.9 Mb. This high-quality assembly enabled the annotation of 41,341 protein-coding genes, 95% of which received functional predictions—providing a foundation for studies of genomic synteny, gene family evolution, and the genetic underpinnings of pathogenesis [7].

Despite this quantum leap, persistent challenges and future frontiers remain. The discrepancy between the k-mer-based genome size estimate (58 Mb) and the final assembly size (148 Mb) underscores the inability of short-read algorithms to resolve such complex repeat architectures. Furthermore, functional annotation remains a considerable hurdle. The discovery that 87.4% of annotated protein-coding genes are monoexonic complicates the ortholog inference and comparative genomics with more intron-rich eukaryotes. Gene ontology (GO) analysis of the KV-1 genome revealed a conspicuous overrepresentation of terms related to metabolic processes, likely reflecting the parasite’s metabolic flexibility. Conversely, the striking underrepresentation of canonical host–parasite interaction domains suggests that *T. foetus* may employ novel or rapidly evolving protein families for pathogenesis, which currently evade standard annotation pipelines [7,12,13].

### 2.2. Comparative Genomics and Genetic Divergence

Whole-genome sequencing (WGS) has been used to study evolutionary pathways and host-specific adaptations of *T. foetus*. WGS data have revealed a genetic divergence between the feline isolate and bovine and porcine strains, allowing better understanding of the parasite’s evolution. While bovine and porcine strains exhibit a close phylogenetic relationship with minimal polymorphism (only 68 SNPs and indels with predicted impact), the number of such differences is orders of magnitude higher when comparing either of them to the feline strain (over 65,000 SNPs and indels). This indicates genetic divergence that likely reflects evolutionary pressures associated with different host environments and tissue tropisms—the gastrointestinal tract in cats versus the reproductive tract in cattle. These genomic comparisons have identified numerous genomic regions exclusive to the feline isolate, which may harbor genes critical for adaptation to the feline intestinal milieu, including mechanisms to cope with bile acids and the gut microbiome. This divergence strongly suggests that the “cat genotype” is not merely a recent host-switch variant but a distinct lineage that has undergone significant evolutionary adaptation. Understanding the functional consequences of these vast genetic differences is a key objective for future research and important for clarifying the taxonomic status of these isolates and whether these isolates represent distinct species or subspecies [2].

Comparative genomic analyses of *T. foetus* isolates from different hosts now allow for the precise identification of gene sets unique to pathogenic or drug-resistant strains [2,7,9]. Such genes, including those encoding metabolic enzymes or factors involved in immune evasion, represent attractive targets for future drug development, as their inhibition could selectively impair parasite survival while sparing the host. Furthermore, the availability of complete, high-quality genome assemblies for diverse isolates facilitates systematic mining of genes encoding surface-exposed and secreted proteins. These molecules, particularly if conserved among pathogenic strains, are promising candidates for next-generation diagnostic assays and recombinant subunit vaccines [14]. In this way, advances in *T. foetus* genomics directly support the rational design of new interventions by enabling the prioritization of molecular targets with functional and clinical relevance.

### 2.3. Metagenomics and Microbiome Interactions: The Parasite Within a Complex Ecosystem

Recent research recognizes *T. foetus* as part of a complex microbial ecosystem—the pathobiome. Metagenomic approaches, particularly 16S rRNA gene sequencing, are opening new investigative avenues by enabling the analysis of the dynamic interplay between *T. foetus* and the host microbiome. A 2023 study profiling the reproductive tract microbiome of bulls infected with *T. foetus* identified over 4300 unique operational taxonomic units (OTUs). The preputial and penile microbiomes of infected animals were dominated by phyla such as Fusobacteria, Firmicutes, Bacteroidetes, Actinobacteria, and Campylobacterota, exhibiting a distinct signature compared to uninfected bulls [15,16].

These observations prompt a fundamental inquiry: does *T. foetus* actively induce dysbiosis, perhaps through the secretion of modulatory factors, or is it an opportunistic pathogen that more effectively colonizes a pre-existing dysbiotic state? Studies by other research groups lend support to the latter hypothesis, demonstrating that some healthy bulls deemed unsuitable for breeding harbored a microbiome similar to that of *T. foetus*-infected individuals. The presence of a core set of phyla suggests a foundational microbiome, whereas shifts at the genus level may represent a dysbiotic signature that facilitates infection. Beyond descriptive profiling, shotgun metagenomics holds significant diagnostic potential by enabling the functional analysis of the entire microbiome. Understanding which metabolic pathways are active within the dysbiotic state may reveal key mechanisms of crosstalk that facilitate the transmission and persistence of *T. foetus* [16].

## 3. Decoding the Transcriptome: From ESTs to Host-Specific Virulence Mechanisms of *Tritrichomonas foetus*

Transcriptomics, the comprehensive analysis of the entire repertoire of RNA transcripts within a cell, provides a foundational framework for understanding the gene expression dynamics and phenotypic plasticity of an organism [17]. In the context of *T. foetus*, transcriptomic investigations have been important, enabling a transition from the static view of the genome to a dynamic understanding of how this parasite adapts and responds to diverse host-derived signals. These studies have progressively revealed the molecular strategies that enable it to inhabit various biological niches (Table 2).

### 3.1. Pioneering Analyses Using Expressed Sequence Tags (ESTs)

The genesis of transcriptomic research on *T. foetus* dates back over two decades to analyses based on Expressed Sequence Tags (ESTs). Although this method is now superseded by modern technologies and was fraught with limitations—such as low sensitivity for weakly expressed genes and difficulty in reconstructing full-length transcripts—it was a pioneering tool during this time [22]. For an organism with an uncharacterized genome, it provided the first, albeit preliminary, catalog of transcriptionally active genes. The first major EST project, which analyzed 5064 clones from a bovine isolate cDNA library, was the first effort to map the parasite’s functional genetic landscape. This initial large-scale sequencing effort assembled the reads into 713 contigs and 1961 singlets, revealing a complex gene expression pattern that reflected the parasite’s adaptations. Crucially, it allowed identification of the “dark transcriptome”—sequences lacking homology to known genes that may represent species-specific sequences of unknown function [18]. Such dark transcriptome sequences in *T. foetus* may, for example, encode novel surface or secreted proteins involved in host tissue adhesion, immune evasion, or modulation of the gut microbiome in the feline or bovine host. For instance, species-specific cysteine proteases or unique variant surface antigens found in RNA-seq and proteomic screens are considered as candidates for previously uncharacterized virulence factors, potentially mediating adaptation to different anatomical niches and influencing pathogenicity. Some of these genes are transcribed only under specific environmental conditions and might play a role in enabling *T. foetus* to colonize diverse hosts or survive immune pressure [6,15].

In this first analysis, over 46% of the unique gene sequences (unigenes) showed no significant similarity to known proteins, underscoring the vast, unexplored territory within the *T. foetus* genome. The transcriptional profile was dominated by genes associated with core cellular processes, particularly translation and ribosomal structure, but also prominently featured genes for anaerobic metabolism (e.g., GAPDH, PEPCK, enolase, and PFOR), demonstrating adaptation to oxygen-poor niches. Detailed exploration of this EST library provided evidence of extensive gene duplication [19]. Numerous genes involved in central carbon metabolism (including multiple forms of PEPCK, GAPDH, and enolase) and those encoding structural proteins (actin, α-tubulin, and β-tubulin) were found to be duplicated, often repeatedly. This may indicate mechanisms similar to those in the related parabasalid *T. vaginalis*, where gene family expansion is thought to generate metabolic flexibility, facilitate immune evasion, and allow for rapid adaptation to fluctuating host conditions. Phylogenetic analyses based on these ESTs suggested that many of these duplication events occurred after the evolutionary divergence of *T. foetus* and *T. vaginalis*, indicating a species-specific expansion of these gene families [19]. The gene families showing the greatest species-specific expansion in *T. foetus* include those encoding enzymes of central carbon metabolism—such as multiple isoforms of phosphoenolpyruvate carboxykinase (PEPCK), glyceraldehyde 3-phosphate dehydrogenase (GAPDH), and enolase—as well as structural proteins like actin, α-tubulin, and β-tubulin. Large gene families encoding cysteine proteases are also duplicated. These expansions may contribute to metabolic versatility, host adaptation, and immune evasion.

Critically, these early studies also uncovered evidence of sophisticated post-transcriptional regulation. Analysis of the 3’ ends of the transcripts revealed that only a small fraction (about 31%) contained the canonical AAUAAA polyadenylation signal (PAS). This strongly indicated a heavy reliance on a multitude of non-canonical signals, with sequences like AGUAAA being highly prevalent. Furthermore, the discovery of numerous alternative polyadenylation (APA) sites suggested that *T. foetus* employs this mechanism for the fine-tuning of gene expression. By modulating the length of the 3’ untranslated region (3’ UTR), APA can influence transcript stability, subcellular localization, and sensitivity to microRNA-mediated regulation. As analogous mechanisms in other protozoan parasites, such as kinetoplastids, are known to regulate key life-cycle transitions, it is highly probable that *T. foetus* leverages APA to dynamically adjust its proteome in response to environmental pressures, such as immune attack or nutrient fluctuations [18,19].

### 3.2. Transcriptional Reprogramming as a Cornerstone of Adaptation and Virulence

High-throughput RNA-sequencing has shown that *T. foetus* modifies its expression profile in response to the host environment through transcriptional reprogramming. While strains isolated from cattle, cats, and pigs are genetically homogenous at the sequence level (sharing > 95% identity), their transcriptional landscapes are divergent, indicating host-specific adaptation. Cysteine proteases (CPs), a vast and functionally diverse family of enzymes, are characteristic of this adaptive strategy. RNA-seq studies have revealed a host-specific pattern of CP gene expression. Feline isolates are characterized by the predominant transcription of the gene encoding cysteine protease CP7, whereas bovine and porcine strains overwhelmingly express the gene for protease CP8. This stark dichotomy at the transcript level may explain the different pathologies observed. The high expression of CP7 in feline isolates is linked to the chronic colitis and persistent inflammation seen in cats. Conversely, the robust expression of CP8 in bovine and porcine strains appears to be a key adaptation to the reproductive tract, where its product likely orchestrates tissue invasion, immune evasion, and ultimately, reproductive failure [6,20,23].

This host-specific specialization extends far beyond this single gene family.

A broader, integrated analysis of RNA-seq data from bovine (BP-4), porcine (PIG30/1), and feline (G10/1) isolates confirmed a profound transcriptomic divergence. Of the top 100 most highly expressed genes, a mere 29 orthologs were shared between the bovine and feline isolates, underscoring the extent of their functional specialization. The parasite’s genomic arsenal includes hundreds of CP-encoding genes—approximately 483 in the bovine strain and 445 in the feline strain—whose differential expression creates distinct secretome profiles [6]. Beyond CPs, the feline isolate exhibits heightened expression of other virulence-associated factors, including surface antigens and BspA-like adhesion proteins, presumed to fortify the parasite’s attachment to the host epithelia [6].

Critically, these investigations offer the first glimpses into the underlying regulatory architecture governing this plasticity. The discovery of numerous actively transcribed genes encoding transcription factors, especially from the MYB family, suggests that unlike some protists that rely heavily on post-transcriptional control, *T. foetus* possesses a dedicated transcriptional machinery to orchestrate complex gene expression programs [24]. Promoter analysis indicates that these MYB proteins may regulate key transcriptional networks governing stress responses and stage-specific gene expression. The confirmed homology of these proteins to R2R3-MYB regulators in other major parasites, such as *T. vaginalis* and *E. histolytica*, places this mechanism within a broader evolutionary context of parasitic adaptation. Collectively, these transcriptomic data indicate that the parasite can reprogram its transcriptome to express host-specific sets of genes [6]. Such approaches—meaning the in-depth analysis of gene expression profiles and elucidation of regulatory architecture (including the participation of MYB factors and host- or strain-specific proteases)—have enabled not only a better understanding of *T. foetus* pathogenesis, but above all, support the development of more targeted therapies. The molecular characterization of the dominant virulence factors present in a given isolate, such as a specific protease or adhesion protein, forms the foundation for the personalization of treatment. Consequently, it becomes possible to design and select targeted drugs (inhibitors) aimed precisely at mechanisms essential for parasite survival or pathogenicity in a particular host species. This approach may improve the therapeutic efficacy and reduce adverse effects [6,24]. Equally important are the possibilities for counteracting the development of drug resistance. Understanding the regulatory mechanisms underlying the adaptive expression of resistance genes (such as switching the expression of proteases, metabolic pathways, or detoxification systems) allows for the prediction and prevention of the emergence of resistant strains. Molecularly targeted therapy may involve not only blocking virulence proteins, but also targeting key transcriptional regulators, thus disabling the parasite’s ability to activate compensatory adaptive pathways. In time, the integration of in vivo expression diagnostics with clinical practice may inaugurate an era of personalized pharmacotherapy for tritrichomonosis, which will not only be more effective but also significantly reduce the problem of secondary drug resistance in livestock and companion animals [6,20].

### 3.3. Regulatory Architecture and Environmental Plasticity

The capacity of *T. foetus* for rapid adaptation is encoded within a unique regulatory architecture. A defining feature of the *T. foetus* genome is its highly streamlined, intron-poor landscape, which dictates that alternative splicing—a major engine of proteomic diversity in higher eukaryotes—plays a minimal, if any, role in its biology. Consequently, the burden of regulatory control is shifted almost entirely to the transcriptional and post-transcriptional levels, including the modulation of mRNA stability and translational efficiency [24]. The identification of a robust repertoire of MYB-family transcription factors provides compelling evidence that *T. foetus* possesses a sophisticated machinery for direct transcriptional regulation. Transcriptome analyses have begun to reveal co-expressed gene modules, or regulons, that are likely co-regulated by these factors. However, these regulatory networks remain putative; their experimental validation through techniques like ChIP-seq or ATAC-seq is a critical next step [6,25]. Furthermore, the role of small RNA pathways in *T. foetus* remains an unexplored frontier. The fact that *T. vaginalis* encodes a functional RNA interference (RNAi) machinery suggests a similar system may exist in *T. foetus* [26].

This regulatory architecture endows *T. foetus* with a remarkable capacity to adapt its physiology in response to environmental insults, a phenomenon best illustrated by its responses to nutrient deprivation and drug pressure. Under conditions of starvation, *T. foetus* can enter a pseudo-dormant state, forming multinucleated, non-dividing forms that undergo rapid multiple fission upon restoration of favorable conditions [27]. While the original studies focused on cell biology, the underlying implication is the activation of a highly orchestrated gene expression program that uncouples DNA endoreplication from cytokinesis. A parallel scenario is anticipated in response to pharmacological pressure. The transcriptional response to nitroimidazole drugs like metronidazole, while not yet systematically mapped, can be inferred from extensive studies in *T. vaginalis*, where resistance typically involves downregulation of prodrug activation enzymes and upregulation of antioxidant systems. Systematic transcriptomic profiling of resistant clinical isolates is a critical priority to elucidate resistance mechanisms and identify novel therapeutic targets [28].

Finally, the emerging area of metatranscriptomics—simultaneous sequencing of host and parasite transcripts—holds immense promise for capturing the molecular crosstalk that occurs during an active infection in vivo.

## 4. Proteomics: From Functional Insights to Novel Therapeutic Targets

Proteomics, the large-scale study of all proteins expressed by an organism, enables analysis of the functional biology of *T. foetus* by characterizing proteins involved in pathogenesis and survival [27]. This approach is crucial for identifying novel diagnostic markers and therapeutic targets because it focuses on the final functional products of gene expression. Table 3 presents the most significant achievements to date in proteomic research on *T. foetus*.

### 4.1. Global Proteome Profiles

High-resolution mass spectrometry has enabled systematic analysis of the *T. foetus* proteome, especially its secreted proteins (the secretome). Initial studies profiling the parasite’s supernatant have identified proteins involved in host–pathogen interactions. Analysis of bovine isolates identified 662 proteins; 121 proteins were detected in all 6 isolates, while the remaining 541 appeared in at least 1 isolate, possibly reflecting adaptation or variation due to culture conditions. Approximately one-third of these secreted proteins were classified as “hypothetical,” lacking known functional annotation. This indicates an uncharacterized set of proteins that may include lineage-specific factors important for parasitism [30].

Bioinformatic analysis showed that nearly half (46.8%) of the secretome proteins were predicted to be of cytoplasmic origin, with additional proteins associated with the plasma membrane (17.2%), mitochondria (11%—likely hydrogenosomes), nucleus (6.9%), and extracellular space (5.7%). This finding has led to debate about the origin of these proteins as to whether these cytoplasmic proteins are secreted via non-canonical pathways (“moonlighting proteins”; as in *Giardia lamblia*) or result from cell lysis during cultivation [31]. The evidence suggests that many have extracellular functions, including modulation of the host immune response [30].

Functional annotation has showed enrichment in proteins with binding (48%) and catalytic (38%) activities, including many proteolytic enzymes, especially cysteine proteases. This research has also identified the immunoreactive secretome—the subset of secreted proteins that elicit a host immune response. Immunodetection assays confirmed that antibodies from infected bulls and immunized mice robustly recognized these secreted proteins across all tested isolates. This immunoreactive repertoire includes well-established virulence-associated proteins. For example, immuno-proteomic assays identified the 65-kDa adhesion protein (AP65) and the heat shock protein Grp78 (BiP) as two of the most potent antigens. AP65 is a classic moonlighting protein, functioning as both a hydrogenosomal enzyme and a surface adhesin, while the stress-inducible chaperone Grp78 can be exposed on the parasite surface to mediate host interactions. Consistent immune recognition of these conserved antigens suggests they could be useful as diagnostic or vaccine targets [14,30]. Recently, a comparative membrane proteomic analysis identified a set of conserved plasma membrane proteins across multiple *T. foetus* isolates, highlighting their potential as universal diagnostic or vaccine targets. This study also included a bioinformatic assessment of antigenicity, suggesting that several of these proteins may be promising candidates for future diagnostic assays or immunoprophylaxis strategies [14]. The consistent immune recognition of conserved secreted antigens, such as AP65 and Grp78, strongly supports their potential as targets for the development of serological diagnostics or subunit vaccines. For example, these proteins may be used as antigens in ELISA assays for diagnostics or as components of subunit vaccines. Preliminary data indicate that antibodies from infected cattle and immunized mice recognize these antigens, supporting their further evaluation. Ultimately, the path from proteomic identification to practical deployment requires comprehensive validation in field and experimental studies, as well as careful optimization of antigen composition to ensure diagnostic sensitivity and specificity under real-world conditions [30].

Although key surface antigens and virulence factors have been identified, translating these findings into veterinary practice will require overcoming several challenges. Foremost among these is the comprehensive validation of selected antigens—such as AP65, Grp78, and conserved membrane proteins—in large-scale, field-based studies, which is essential to determine their sensitivity and specificity under real-world conditions. It is also necessary to assess antigenic variation among field isolates to ensure reliable detection across different strains and to maximize the protective efficacy of prospective vaccines. Further, effective immunoprophylaxis depends not only on immunogenicity but also on the durability of protection and the practical aspects of antigen production and formulation for commercial use. On the diagnostic front, converting proteomic discoveries into robust, rapid, and affordable tests—such as ELISA or lateral flow assays—demands optimization for deployment in varied veterinary settings. Additionally, large-scale clinical validation in diverse geographic and host populations remains a prerequisite for regulatory approval and widespread adoption. Integrating these proteomic advancements with innovative technologies, such as multiplexed antigen panels or peptide fingerprinting, offers a promising path forward but will need further cost–benefit analysis and field implementation. Despite these translational hurdles, accelerating progress in omics-driven validation, bioinformatics, and cross-disciplinary collaboration continues to bring the practical deployment of novel diagnostics and subunit vaccines for tritrichomonosis closer to reality. Addressing these application-oriented considerations will markedly enhance the impact of proteomic research and facilitate the introduction of effective tools for disease control in both livestock and companion animals.

### 4.2. Molecular Mechanisms of Virulence and Host-Interaction

Proteomic studies show that the pathogenicity of *T. foetus* involves effector proteins, including cysteine proteases (e.g., CP8/CP30) and the 65-kDa adhesion protein (AP65). Cysteine proteases (CPs) play major roles at multiple stages of host–parasite interactions. The primary role of CPs begins with adhesion, a process that can be abrogated by specific inhibitors like E-64. This is exemplified by the protease CP8 (CP30), which is crucial for adhesion in feline isolates and known for its potent cytopathic effects in the bovine host. After adhesion, CPs can degrade extracellular matrix components, affect the mucus barrier, and neutralize host immunoglobulins [29,32,33].

This host-specific adaptation extends to the parasite’s core cellular machinery. Comparative proteomics has revealed differential expression of cytoskeletal proteins, suggesting that alterations in cell structure and motility are crucial for niche specialization. For instance, coronin, an actin-regulating protein, is more abundant in feline isolates, likely enhancing motility in the dynamic intestinal environment. Conversely, centrin, a protein involved in cell division, is more prevalent in bovine strains, perhaps reflecting adaptation for intense replication in the bovine reproductive tract [29].

Beyond the secretion of individual proteins, *T. foetus* likely employs a more strategic mode of communication: the release of extracellular vesicles (EVs). These “virulence packages” can deliver a concentrated cargo of proteins and nucleic acids, and their characterization represents a critical frontier in understanding the full complexity of its pathogenic strategy [34,35].

### 4.3. Proteomics as a Driver for Drug Discovery

Proteomics has enabled the identification of proteins essential for parasite survival and pathogenesis, providing potential targets for new therapies. Early studies identified a group of proteins with therapeutic potential, including cysteine proteases (CPs), which are also virulence factors. Proteomic analyses have identified at least 15 distinct CPs in bovine *T. foetus*, many of which were previously uncharacterized, offering a wide array of potential targets for inhibition [29]. This therapeutic strategy has been supported by studies using specific CP inhibitors. For example, vinyl sulfone inhibitors such as K11777, which are designed to target parasite CPs, have been shown to significantly reduce the cytotoxic effects of *T. foetus* on host cells in vitro. Furthermore, pre-treatment of parasites with these inhibitors diminished genital colonization in a murine model, providing crucial proof-of-concept that targeting CPs is a valid therapeutic approach [36,37]. Recent data support further evaluation of cysteine protease (CP) inhibitors as potential therapeutics for *T. foetus* infections. Given the central role of CPs in key virulence processes—including tissue invasion, immune evasion, and cytotoxicity—inhibiting these enzymes could directly disrupt pathogenicity and parasite survival. Vinyl sulfone inhibitors (e.g., K11777) reduced *T. foetus* cytotoxicity in vitro and decreased colonization in animal models, confirming proof-of-concept for this strategy [29,38]. However, challenges remain before clinical application. These include ensuring the selectivity of CP inhibitors for parasite versus host proteases to avoid adverse effects, maintaining the durability of therapeutic response in the face of potential adaptive mechanisms, and mitigating the risk of resistance development, particularly considering the large and redundant repertoire of CP-encoding genes in *T. foetus*. Ongoing research should thus prioritize the structural optimization of inhibitors, exploration of synergistic drug combinations, and monitoring of therapeutic impacts via proteomic methods [29,38].

Furthermore, proteomics has highlighted crucial enzymes in metabolic pathways that are absent in the host or sufficiently divergent to allow for selective targeting. The best example is inosine monophosphate dehydrogenase (IMPDH), a rate-limiting enzyme in the de novo biosynthesis of guanine nucleotides. Since *T. foetus* relies heavily on purine salvage, IMPDH was identified early on as a promising target. Inhibition of this enzyme with compounds like mycophenolic acid and ribavirin disrupts parasite growth. High-resolution crystal structures of *T. foetus* IMPDH have revealed key differences in the active site compared to mammalian homologs, providing a structural basis for the design of parasite-specific inhibitors [39,40]. The parasite’s ability to develop resistance to IMPDH inhibitors has also been studied, revealing a novel mechanism where resistant strains downregulate hypoxanthine transport and upregulate xanthine salvage, effectively creating a metabolic bypass around the drug-induced block [41].

Another critical area is targeting mechanisms of drug resistance, particularly to nitroimidazoles like metronidazole. The primary mechanism of metronidazole resistance in trichomonads involves the impaired reductive activation of the drug within the hydrogenosome, often due to the downregulation or loss of key enzymes like pyruvate/ferredoxin oxidoreductase (PFOR) [42]. While direct proteomic data on drug-resistant *T. foetus* is sparse, studies on resistant mutants have confirmed the loss of hydrogenosomal pyruvate metabolism, which is compensated by an increased rate of glycolysis. This metabolic shift suggests that enzymes in the compensatory cytosolic pathways, such as pyruvate decarboxylase (PDC), could serve as secondary targets to overcome resistance [16].

Finally, proteomic identification of ornithine decarboxylase (ODC), a pivotal enzyme in polyamine biosynthesis, has opened another promising therapeutic avenue. Polyamines are essential for cell proliferation and differentiation in virtually all organisms, including parasites. Crucially, studies have shown that inhibition of ODC in *T. foetus* results not only in growth arrest but also leads to the destruction of hydrogenosomes and a decrease in the abundance of key hydrogenosomal enzymes. This remarkable finding links polyamine metabolism directly to the integrity of a vital organelle, suggesting that ODC inhibitors could deliver a multi-pronged attack on the parasite’s viability [18].

### 4.4. Subcellular Proteomics: Uncovering Unique Structural Markers

A significant advance in *T. foetus* proteomics has been the characterization of proteins associated with unique subcellular structures, offering highly specific diagnostic and therapeutic targets. A prime example is the proteomic analysis of the costa, a prominent striated fiber unique to trichomonads [18]. An analysis of an enriched costa fraction identified several novel proteins, with costain 1 being the most abundant, establishing it as the first molecular marker for this organelle [43]. Given that the costa is absent in host cells, its constituent proteins represent exceptionally promising and specific targets for diagnostics or therapies that could interfere with the parasite’s structural integrity without affecting the host.

### 4.5. Metaproteomics: Interrogating In Situ Interactions

Metaproteomics expands *T. foetus* studies from in vitro cultures to analysis within the host environment. As a discipline dedicated to the global characterization of proteins in environmental samples, metaproteomics provides the unique capacity to simultaneously profile the proteomes of the parasite, the associated microbiome, and the host, thereby capturing the dynamic molecular dialog that unfolds in situ at the site of infection.

Metaproteomic analysis of clinical specimens such as vaginal mucus or fecal samples could provide new insights into pathogenesis. It would enable not only the identification of parasite-specific virulence factors as definitive evidence of active infection but also the characterization of the host’s proteomic response signature. A recent microbiome study on bulls found changes in protein composition of seminal plasma during *T. foetus* infection, supporting further biomarker research [16,41].

Furthermore, metaproteomics serves as a critical functional complement to metagenomics; while metagenomics answers the question “who is there?” by cataloging the genetic potential of a microbial community, metaproteomics answers the question “what are they doing?” by delineating the active metabolic pathways and protein functions.

From a diagnostic standpoint, this approach holds dual potential. In an initial discovery phase, untargeted or “shotgun” metaproteomics can identify previously unknown protein markers. Subsequently, based on these findings, highly sensitive and specific assays can be developed using targeted proteomics. This technique has already been successfully applied to detect *T. foetus* in preputial smegma from bulls by identifying its species-specific peptide “fingerprints” [44]. This method holds a key advantage over PCR-based tests as it detects proteins, which provides evidence of viable, metabolically active parasites, not merely their residual DNA. As the sensitivity of mass spectrometers continues to improve, such direct protein detection could become a powerful diagnostic tool, supplementing PCR by providing information not only on the presence but also on the activity of the pathogen.

Challenges include the complexity of clinical samples and assigning peptides to their organism of origin, but advances in technology and databases are expected to improve these analyses.

## 5. Future Directions: Charting the Next Decade of *T. foetus* Research

Omics research on *T. foetus* over the past two decades has improved understanding of its biology and pathogenesis. However, knowledge gaps remain, especially regarding genome completeness, gene regulation, protein function, and host–parasite interactions. Addressing these issues will require pangenomic studies, functional genomics, and integrative multi-omics approaches to identify new biological mechanisms and therapeutic targets.

### 5.1. Advancements in Genomics: From Complete Genomes to Population-Level Insights

Despite progress in sequencing and assembly, current *T. foetus* reference genomes are still incomplete, especially in repetitive regions, limiting their utility. Obtaining complete, telomere-to-telomere genome assemblies is a priority to support studies of gene evolution and host adaptation.

A current priority is to obtain complete, high-quality genome sequences of *T. foetus* isolates from cats and pigs. To date, genomic data for these isolates have been generated mainly through short-read sequencing technologies, using mapping strategies against the bovine reference genome [2]. While this approach is suitable for initial comparative studies, it does not allow for the full reconstruction of unique sequences, structural rearrangements, or host-specific genes. Therefore, de novo genome sequencing of feline and porcine isolates using long-read technologies (e.g., PacBio HiFi or Oxford Nanopore) is needed, as these methods enable the generation of continuous, reference-grade assemblies. This is especially relevant given the occurrence of *T. foetus* infections in cats. Access to such genomic resources would facilitate in-depth analyses of gene families, duplications, and regulatory elements [45,46].

Furthermore, a substantial proportion of predicted genes in existing genomes remain annotated as “hypothetical proteins” [9]. A major future direction must be the intensification of functional annotation efforts, integrating transcriptomic and proteomic datasets and implementing advanced gene function screening tools, such as CRISPR-based approaches, to elucidate the biological roles of these uncharacterized proteins. Ultimately, generating these high-quality, well-annotated genomes across multiple isolates will pave the way for population genomics. Sequencing a significantly larger number of isolates from diverse hosts and geographic regions will be pivotal to resolving ongoing debates regarding whether these isolates represent distinct genotypes within a single species or constitute cryptic, genetically distinct species, providing fundamental insights into the parasite’s epidemiology and evolutionary dynamics.

### 5.2. Expanding Transcriptomic Analyses: From Bulk RNA-Seq to Single-Cell and In Vivo Insights

Future transcriptomic investigations should move beyond descriptive analyses to address key technical challenges and explore currently uncharacterized regulatory mechanisms. One important technical obstacle arises from the parasite’s highly repetitive genome, which complicates the accurate mapping of sequencing reads, particularly for large gene families such as cysteine proteases and surface antigens. As a result, it remains challenging to determine which specific family members are being regulated. Additionally, most existing studies have focused primarily on messenger RNA, with relatively little attention given to non-coding RNAs (ncRNAs) in *T. foetus*. Systematic analyses of small ncRNAs and long ncRNAs, which are known to play regulatory roles in other parasites, are therefore needed [19].

To overcome these limitations, future research should utilize single-cell RNA sequencing (scRNA-seq). This approach will be useful for analyzing population-level heterogeneity and for identifying rare cell subpopulations that may be involved in processes such as drug resistance or dormancy. Additionally, to better understand the interaction between the parasite and its host, in vivo metatranscriptomics is an important next step. Analyzing dual RNA-seq data from clinical samples enables simultaneous profiling of host and parasite gene expression, revealing the molecular interactions that occur during infection. Alongside these exploratory strategies, targeted transcriptomic studies under controlled conditions—such as exposure to therapeutic drugs or co-culture with host immune cells—are important for dissecting specific response pathways and for mapping the regulatory networks that contribute to the adaptability and virulence of *T. foetus* [27].

### 5.3. Advancing Proteomics: From In Vivo Profiling to Functional Validation

While proteomic studies have expanded knowledge of *T. foetus*, several gaps remain. One limitation is that current datasets provide incomplete proteome coverage, likely missing low-abundance regulatory proteins and those expressed only in vivo. As a result, the actual in vivo proteome of *T. foetus*—the set of proteins produced within the host—is still largely uncharacterized and remains an important focus for future context-specific studies using methods such as metaproteomics. Another challenge involves determining the functions of many “uncharacterized” proteins. Since approximately one-third of the secretome proteins do not have assigned roles, functional genomics approaches will be necessary to clarify their biological significance.

Furthermore, the landscape of post-translational modifications (PTMs), which are important for regulating protein function and virulence, remains largely unexplored. Advanced proteomic techniques will be needed to investigate PTMs such as phosphorylation and glycosylation to clarify their roles in signaling and immune recognition.

The mechanisms of intercellular communication and protein cooperation also require further characterization. In *T. vaginalis*, extracellular vesicles (EVs) function as carriers of virulence factors. It is likely that *T. foetus* also releases such vesicles; identifying their protein cargo could help explain how groups of virulence factors are delivered to host cells [42].

Finally, there is a challenge in translating proteomic discoveries into practical applications. While some promising candidates for vaccines and diagnostics have been proposed, the process from protein discovery to validated product requires interdisciplinary collaboration, thorough in vivo validation, and planned development.

### 5.4. Integrative Multi-Omics and Systems Biology: A Holistic Future

A future direction for research is the integration of data from different omics approaches into a systems-level understanding of *T. foetus* biology. Multi-omics studies that combine genomic, transcriptomic, and proteomic data from the same biological samples can support the construction of comprehensive regulatory and metabolic network models. The application of machine learning and artificial intelligence (AI) to these large integrated datasets may help to identify complex patterns, predict gene function, and uncover therapeutic targets not evident from any single omics layer.

Recent advances in veterinary molecular parasitology suggest that more personalized strategies for treating *T. foetus* infections are possible. Integrating genomic, transcriptomic, and proteomic data about a given infection—so-called “omics-based stratification of treatment”—can help identify molecular signatures relevant for drug susceptibility and virulence, guiding therapeutic choices. In the future, routine use of metaproteomic monitoring, such as analysis of the secretome or specific resistance-related proteins in clinical samples (for example, feces from cats or genital mucus from cows), could allow real-time tracking of treatment effectiveness. For instance, sequential mass spectrometry of parasite peptides could provide evidence of the elimination of virulence factors or the emergence of resistance markers, helping to inform clinical management and early intervention [6]. An omics-guided combination therapy approach is also being explored as a strategy to address drug resistance. Insights from analyses of regulatory and metabolic networks, revealed by comparative proteomics and transcriptomics, may support the rational design of therapies targeting multiple pathways. For example, such treatments could combine inhibitors of cysteine proteases, essential metabolic enzymes (like IMPDH), and pathways associated with resistance, potentially reducing the risk of rapid emergence of resistant strains [6]. Implementation of these strategies will require further technical advances and robust validation in field studies, but they may improve the control of tritrichomonosis and limit the development of drug resistance.

## Figures and Tables

**Table 1:** Key achievements in *Tritrichomonas foetus* genomics.

Strain(s)	Main Achievement	Genomic Parameters	Biological Conclusions	Study (Year)
K (bovine)	First draft genome assembly	- Genome size: 68.5 Mb - Contigs: 3730 - Genes: 25,353 - Functional annotation: ~31%	- Highly fragmented genome - 47% of proteins annotated as “hypothetical”	Benchimol et al. (2017) [9]
TF1 (bovine) TF2 (feline) TF3 (porcine)	First comparative WGS analysis of three host strains	- Moderate/high-impact variants (vs. TF1): TF2: 65,569 and TF3: 68 - Homologous variants vs. strain K: TF1: 39,784, TF2: 338,632, TF3: 38,916	- 964× higher divergence for TF2 vs. TF1/TF3 - TF2 represents a separate evolutionary lineage	Dąbrowska et al. (2020) [2]
KV-1 (bovine)	First chromosome-level genome assembly	- Repeat-rich genome (62% repeats) resolved - N50 improved to 22.9 Mb - reference for future functional studies	- Repeat-rich genome (62% repeats) resolved -N50 improved to 22.9 Mb - reference for future functional studies	Abdel-Glil et al. (2024) [7]

**Table 2:** Key transcriptomic achievements and parameters in *Tritrichomonas foetus*.

Isolate(s) (Host)	Main Focus/Achievement	Main Virulence/Functional Gene Families Identified	Key Biological Insights	Study (Year)
KV-1 (bovine)	First large-scale EST and proteome profiling and functional annotation	- glycolytic enzymes - actin/tubulin - CPs - adhesins (AP65-1) - ribosomal proteins	- translation and energy metabolism genes dominate - proteome validates transcriptome - novel chemotherapeutic targets	Huang et al. (2013) [18]
K1 (bovine)	Proteomic and transcriptomic analysis of hydrogenosome-enriched fractions	- hydrogenosomal enzymes - energy metabolism - iron-sulfur proteins - CPs	- functional validation of hydrogenosomal proteins - link between transcriptome and organellar proteome - metabolic adaptation	Oyhenart et al. (2014) [19]
BP-4 (bovine), G10/1 (feline)	Comparative transcriptomics and in silico drug-target identification	- CPs - metabolic enzymes - surface antigens - hypothetical proteins	- similarities in drug-targets - host-specific expression of virulence factors	Morin-Adeline et al. (2014) [20]
BP-4 (bovine), G10/1 (feline), PIG30/1 (porcine)	Comparative RNA-seq of three isolates and host-specific gene expression	- CPs - BspA-like - tetraspanins - MYBs	- feline: dominant CP7 expression - bovine/porcine: CP8 - host-specific virulence adaptation - gene duplication - MYB-driven regulation	Morin-Adeline et al. (2015) [21]
BP-4 (bovine), G10/1 (feline), PIG30/1 (porcine)	Comparative transcriptomics, feline adaptation, and virulence factors	- MYB TFs (552 total, 92 highly expressed in G10/1) - BspA-like, tetraspanins - papain-like, calpain-like - subtilisin-like - GP63-like	- feline isolate (G10/1): unique profile - more virulence factors especially MYB and BspA-like proteins - MYB motifs in promoters	Alonso et al. (2022) [6]

**Table 3:** Key proteomic achievements in *Tritrichomonas foetus* research.

Isolate(s) (Host)	Proteomic Focus	Key Findings	Biological/Clinical Implications	Study (Year)
Bovine (KV-1)	Global proteome profiling	68 abundant proteins (2-DE/MS); dominant: glycolytic enzymes (GAPDH, PEPCK), cysteine proteases (15 isoforms, TfCP8 most abundant), adhesins (AP65-1)	Chemotherapeutic targets; reference proteome map	Huang et al. (2013) [18]
Bovine vs. feline	Comparative proteomics	24 proteins with ≥4-fold differential expression; clear differences in cysteine protease (CP) profiles; higher CP activity in feline isolates	CPs as key virulence determinants; basis for vaccine/drug development	Stroud et al. (2017) [29]
6 bovine isolates	Secretome (secreted proteins)	662 proteins in supernatant; 121 core proteins (all isolates); Ap65 and Grp78: immunodominant antigens; 32.9% “hypothetical” proteins	Candidates for diagnostics/subunit vaccines; immunomodulatory potential	Abdala et al. (2023) [30]
Various bovine isolates	Membrane proteomics	85 core membrane proteins; enrichment in sialidases, transmembrane proteins, hydrolases; high antigenic potential	New diagnostic and vaccine targets; sialidases as virulence factors	Rivero et al. (2024) [14]

## Data Availability

The data presented in this study are available on request from the corresponding author.
